# Copper induce zebrafish retinal developmental defects via triggering stresses and apoptosis

**DOI:** 10.1186/s12964-020-00548-3

**Published:** 2020-03-14

**Authors:** Guang Zhao, HaoJie Sun, Ting Zhang, Jing-Xia Liu

**Affiliations:** College of Fisheries, Key Laboratory of Freshwater Animal Breeding, Ministry of Agriculture, Huazhong Agricultural University, Wuhan, 430070 China

**Keywords:** ROS, ER, Apoptosis, Retina, *cox17*, *atp7a*

## Abstract

**Background:**

The disorder of copper homeostasis is linked with disease and developmental defects, and excess copper_nanoparticles (CuNPs) and ion (Cu^2+^) will induce developmental malformation and disease in organisms. However, little knowledge is available regarding its potential regulation mechanisms, and little study links excess copper with retinal developmental malformation and disease.

**Methods:**

Embryos were stressed with copper (CuNPs and Cu^2+^), and cell proliferation and apoptosis assays, reactive oxygen species (ROS) and endoplasmic reticulum (ER) signaling detections, and genetic mutants *cox17*^−/−^ and *atp7a*^−/−^ application, were used to evaluate copper induced retinal developmental malformation and the underlying genetic and biological regulating mechanisms.

**Results:**

Copper reduced retinal cells and down-regulated expression of retinal genes, damaged the structures of ER and mitochondria in retinal cells, up-regulated unfold protein responses (UPR) and ROS, and increased apoptosis in copper-stressed retinal cells. The copper induced retinal defects could be significantly neutralized by ROS scavengers reduced Glutathione (GSH) & N-acetylcysteine (NAC) and ER stress inhibitor 4- phenylbutyric acid (PBA). Blocking the transportation of copper to mitochondria, or to trans-Golgi network and to be exported into plasma, by deleting gene *cox17* or *atp7a*, could alleviate retinal developmental defects in embryos under copper stresses.

**Conclusions:**

This is probably the first report to reveal that copper nanoparticles and ions induce retinal developmental defects via upregulating UPR and ROS, leading to apoptosis in zebrafish embryonic retinal cells. Integrated function of copper transporter (Cox17 and Atp7a) is necessary for copper induced retinal defects.

**Graphical abstract:**

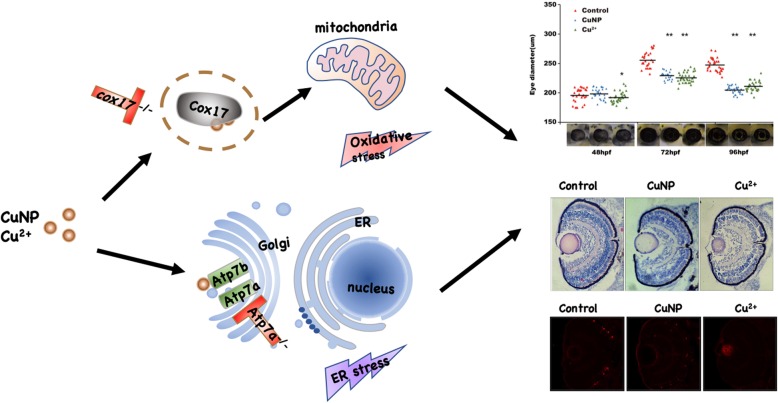

## Background

Copper is an essential trace element with crucial roles in cell activities during organism growth and development [1, 2]. However, excess copper is harmful to cells, leading to protein damage and reduced cell proliferation [3, 4]. Many diseases are associated with the accumulation of copper in cells, such as Wilson disease (WD), Menkes disease (MD), and Eales diseased (ED) [5–9]. ED is an idiopathic inflammatory disease especially prevalent in the Indian subcontinent [10], and patients with either MD and WD develop retinal degeneration [11]. However, little is known about how copper overload in cells induces retinal disease.

As a special form of copper, copper_nanoparticles (CuNPs) are widely applied in many fields due to their unique physiochemical properties, such as small size and antibacterial activity [12–14]. CuNPs have been reported to cause gill injury and acute lethality in adult zebrafish [15–17], and cause zebrafish embryonic developmental defects, such as short body length and dysfunctional locomotor behavior [18, 19]. Additionally, it has been reported that copper ions (Cu^2+^) mediate most of the biological functions of CuNPs during fish embryogenesis [18]. However, there have been few reports on the damage of CuNPs and Cu^2+^ to the embryonic retinal development in vertebrates as well as their potential molecular mechanisms.

The dysfunction of critical regulatory signals not only causes abnormal embryonic development but also induces adult diseases. Mutations in *CERKL*, *SEC23A*, and *SEC13* genes can cause the development of human retinal degenerative diseases. Similarly, zebrafish mutants of these genes also show retinal developmental defects during embryogenesis [20–22], suggesting conserved functions of the important signaling and factors exist in two biological diverse models. However, no cases have been linked copper overload with retinal diseases in the absence of an underlying disorder model [8].

It was reported that copper induced reactive oxygen species (ROS) in vertebrates, which consists of a series of natural byproducts generated by cells during metabolism and plays important roles in cell signaling and homeostasis [23]. ROS generated by excess copper can cause cell damage [24]. Copper led to isolated rat liver mitochondria impairment and caused DNA damage in zebrafish ZFL cells by its induced ROS [25, 26]. Additionally, copper-induced ROS can cause endoplasmic reticulum (ER) stress in cultured cells and patients with Wilson disease [4]. Abnormal protein accumulation is one of the conditions that induce ER stress, which activates the unfolded protein response (UPR) to ensure the high fidelity of these abnormal proteins [27]. Studies revealed that ER stress induced retinal degeneration in rat models of autosomal dominant retinitis pigmentosa (ADRP) caused by the dysregulation of calcium homeostasis, and ADRP is also considered to be related to mitochondrial dysfunction [28, 29]. The aformentioned studies are almost performed in cell lines, however, it is still unclear whether ROS and ER are involved in copper-induced retinal developmental defects in vertebrate model zebrafish.

The trafficking of copper in cells has been clearly illuminated, and the normal trafficking of copper is crucial for cell metabolism homeostasis. Copper is transported across the plasma membrane by Ctr1 (*slc31a1*, solute carrier family 31 member 1 in ZFIN), being transported to mitochondria by Cox17, and enters the circulation by the pumping action of copper ATPase7a (ATP7a) which resides in Trans-Golgi network (TGN) [30, 31]. The functional abnormalities of chaperones Cox17 and Atp7a tend to cause serious diseases in humans [32, 33]. Meanwhile, rare studies have been performed about CuNPs trafficking in cells as well as the correlation of different copper chaperones with their induced retinal defects in in vivo model zebrafish.

In order to reveal the potential mechanisms of copper (CuNPs and their released Cu^2+^) in inducing retinal deformities, zebrafish embryos were treated with CuNPs and Cu^2+^ as described in our previous studies [18, 19], and the expressions of retinal genes and genes associated with ROS and ER stress sensors were tested first, followed by the examination of the neutralization effects of ROS scavengers reduced Glutathione (GSH) & *N*­acetylcysteine (NAC) and ER stress inhibitor 4- phenylbutyric acid (PBA) on copper induced retinal defects. Moreover, *cox17*^*−/−*^ and *atp7a*^*−/−*^ mutants were used to verify the correlations between the integral function of copper trafficking chaperones and the occurrence of developmental defects in copper-stressed embryos.

## Materials and methods

### Fish lines, reagents, and antibodies

The AB wild type (WT), and *cox17*^−/−^ [34, 35] and *atp7a*^−/−^ (prepared in our lab, and the transcriptional profiles of the mutated embryos were organized in another manuscript) adult zebrafish were cultured in a circulating filtration system (28 ± 0.5 °C, 14,10 h light: dark). In this study, *cox17*^−/−^ and *atp7a*^−/−^ were used to test their integral function in copper induced embryonic retinal defects. Natural eggs were obtained and maintained at 28.5 °C in an incubator. The ages of the embryos and larvae were expressed by hours post-fertilization (hpf) or days post-fertilization (dpf). The following reagents and antibodies were used in the study: copper sulfate, copper nano-powders and PBA (Sigma-Aldrich, USA); NAC, reduced GSH, and RIPA lysis (Beyotime, China), and TRIzol (Life Technology Co, USA). Antibodies: Zpr-1, Zpr-3, red opsin, blue opsin, and green opsin (Abgent, USA); PDI, p­eIF-2α, and XBP1-s (Huaan Biotechnology, China), phospho-H3 (PH-3) (Cell Signaling Technology, USA).

### Ethics statement

All animals and experiments were conducted in accordance with the “Guidelines for Experimental Animals” approved by the Institutional Animal Care and Use Ethics Committee of Huazhong Agricultural University (permit number HZAUFI-2016-007).

### Embryo treatment and phenotype observation

The preparation of copper (CuNPs and Cu^2+^) referred to the methods in our previous study [18]. The embryos were exposed to copper before sphere stage [36]. Copper, including CuNPs and Cu^2+^, with a concentration of 3.9 uM was used for all copper stress experiments in this study. NAC (200 μM) and GSH (100 μM) were added 2 h before copper treatment. PBA (50 μM) was added at 24 hpf. The embryos were collected at the indicated stages. Embryos from the control and the treated groups were observed and photographed under light microscopy (Leica M205FA) to examine their embryonic morphology. Embryos were photographed and the diameters of the embryonic eyes were calculated by the scale generated by the microscopy software.

### Transmission electron microscopy (TEM)

The zebrafish embryos, copper (CuNPs and Cu^2+^) stressed WT, *cox17*^*−/−*^, and *atp7a*^*−/−*^, with their control respectively, were collected at 96 hpf and fixed with 2% glutaraldehyde for 4 h at 4 °C. Then, the embryos were washed 3 times with 0.1 M PBS (pH 7.4) and were transferred to 1% osmic acid at room temperature (20 °C) for 2 h. The embryos were dehydrated in a graded ethanol solution, and embedded in Quetol 812 (Nisshin EM Co., Ltd.; Tokyo, Japan) for sectioning. Finally, a transmission electron microscope (Hitachi H-7650 TEM Japan) was used to acquire the images. The structure of organelle mitochondrion and endoplasmic reticulum in retinal cells in copper stressed embryos and their controls were tested in this study.

### Frozen section

Embryos at 72 hpf, 96 hpf, and 10 dpf were fixed with 4% PFA overnight at 4 °C, and then were dehydrated with 30% sucrose PBS solution for 2 h at room temperature. Next, the permeated embryos were embedded in Tissue-Tek® O.C.T. compound (Sakura Finetek, USA) for cryosectioning at 6 μm ~ 8 μm in thickness with frozen microtomy (Thermo scientific, USA). After drying at 4 °C, the sections were stored for H&E staining, immunofluorescence, and TUNNEL assays.

### Hematoxylin and eosin (H&E) staining

The H&E staining was performed as reported previously [21]. Briefly, after being washed quickly with distilled water, the cryosections were stained with filtered 0.1% hematoxylin solution for 2 min at room temperature (RT). Following a quickly wash in distilled water, the sections were treated with 1% acid alcohol for 30 s, washed with water for 4 min, and then counterstained in 0.5% eosin for about 30 s at RT. After being washed with water for 3 min, the sections were gradually dehydrated in 95 and 100% ethanol and then in xylene three times for 15 min each. Finally, high-resolution images for the H&E staining sections were obtained under a microscope (ZEISS Axio Imager A2).

### DCFH-DA assay

In this study, the ROS levels in embryonic retina after copper stresses were measured with A DCFH-DA (2′, 7′-Dichlorodihydrofluorescein-diacetate) Reactive Oxygen Species Assay Kit (Beyotime, China) following the protocol. The embryos were counted and photographed under Stereoscopic Microscope (Lecia M205FA).

### RNA preparation and real-time PCR analysis

Quantitative PCR was used to detect the expression of the target genes. Total RNA was extracted with TRIzol Reagent, and the cDNA was synthesized by using an M-MLV Reverse-Transcript Kit (Applied Biological Materials Inc., BC, Canada). Real-time qPCR was performed using iQ™ SYBR® Green Super mix (Bio-Rad Laboratories, Hercules, CA, USA) in a CFX Connect™ Real-Time PCR Detection System (Bio-Rad Laboratories, Hercules, CA, USA). Differences were calculated by the ΔΔCt comparative quantization method using β-actin as an internal control, and the data were analyzed with one-way analysis of variance (ANOVA) and post hoc Tukey’s test (**, *P* < 0.01; *, *P* < 0.05). The specificity of all primers was tested before use, and the sequences of these primers are listed in Table S1.

### Whole-mount in situ hybridization (WISH)

WISH was performed as described in our previous study [19]. Gene-specific primers were designed based on available information, and the PCR products were cloned into pGEM-T Easy vectors for anti-sense RNA probe synthesis. The primers are listed in Table S1. The anti-sense RNA probes labeled with digoxygenin (Roche Diagnostics) were used to verify the expression of target genes in whole-mount embryos. The images were taken under a Leica microscope (Leica M205FA, Germany).

### Western blot

Embryos at 72 hpf were homogenized and transferred to RIPA lysis buffer with proteinase inhibitor. Then, the appropriate SDS-PAGE loading buffer was added and the obtained protein was boiled for 10 min. An almost equal amount of protein in each line was separated by polyacrylamide gel electrophoresis. The separated protein was transferred to polyvinylidene fluoride microporous membrane (Bio-Rad Laboratories, Hercules, CA, USA). The blots were blocked with 0.2% skim milk in TBS containing 0.1% Triton X-100, followed by incubation first with the primary antibodies (1:200), and then with secondary antibodies (1:1000). Finally, the blots were visualized using enhanced chemiluminescence (Bio-Rad Laboratories, Hercules, CA, USA).

### Immunofluorescence and TUNNEL assay

The immunofluorescence was performed with the primary antibodies against Caspase3, PH-3, Zpr-1, Zpr-3, red opsin, rod opsin, blue opsin, and PDI, were used in a 1:200 dilution. Secondary antibodies conjugated with Alexa Fluor 488 and 599 were used in a 1:500 dilution. 40, 6-diamidino-2-phenylendole (DAPI) was used to label nuclei. The apoptosis detection was performed with a TUNNEL detection kit (Vazyme, Nanjing, China) following the protocol. The images of immunofluorescence and apoptosis were obtained with a confocal microscope (Olympus FV1000 Confocal Microscope, Japan).

### Statistical analysis

The sample size for different experiments in each group was larger than 10 embryos (*n* > 10) with 3 biological replicates for each test. The statistical data of in situ hybridization were determined by hypergeometric distribution analysis using the software of R-console. The other data were analyzed by Student’s unpaired 2-tailed t-test on GraphPad Prism 8.2.1. Statistically significant differences among groups were indicated by ** *P* < 0.01, and * *P* < 0.05 and the effect size (R squared) was marked above the column or the sample dot.

## Results

### Excess copper causes small eyes in zebrafish embryos

**Fig. 1:**
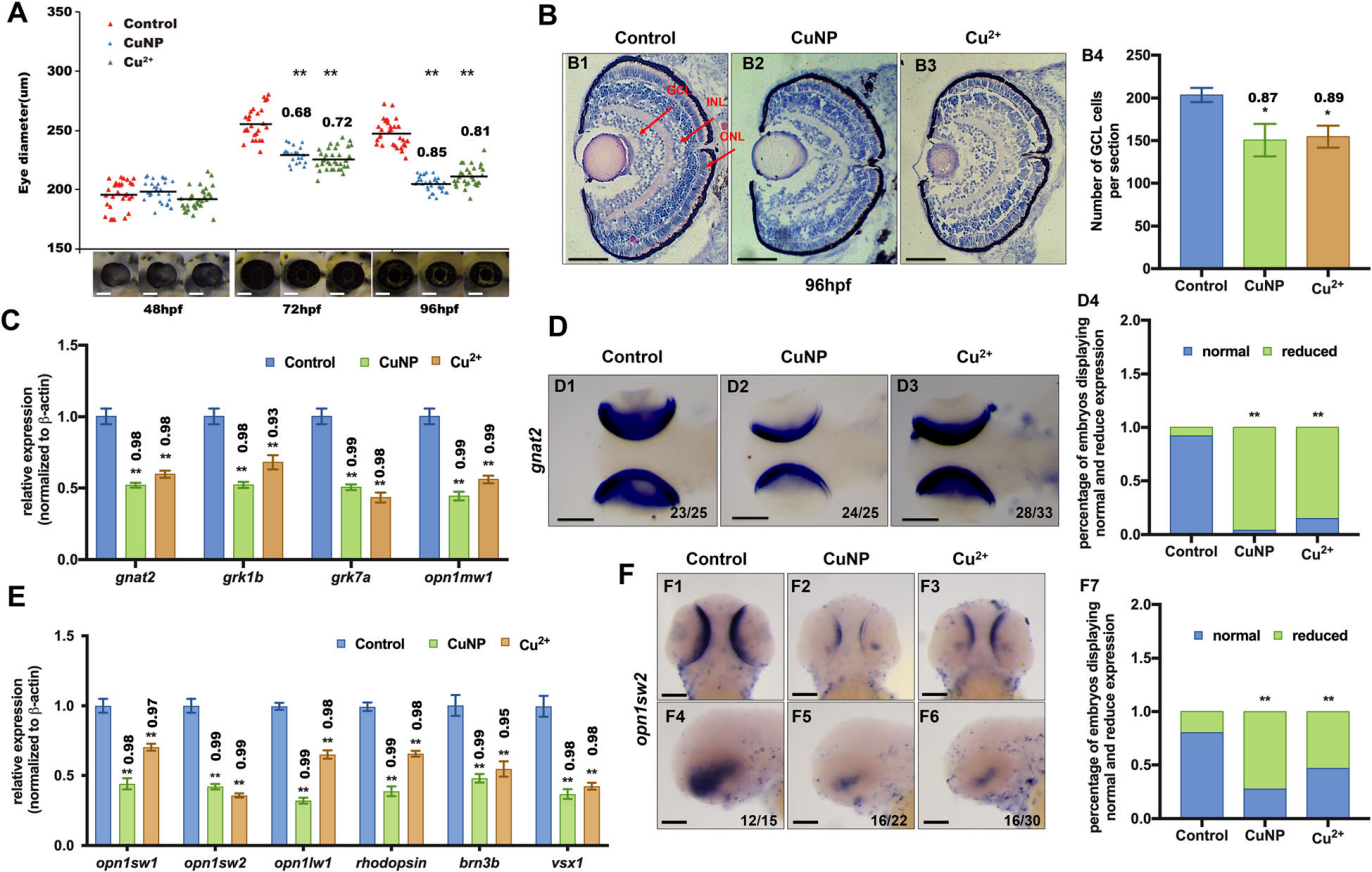
Retinal developmental defects in copper stressed embryos. **A** Measurement of eye diameter of the embryos from control, Cu^2+^- and CuNPs- stressed groups at 48 hpf, 72 hpf, and 96 hpf, respectively. **B** H&E staining analysis of retina of embryos from control (**B1**), Cu^2+^-stressed (**B2**) and CuNPs-stressed groups (**B3**) at 96 hpf. Control embryos at 96 hpf developed normal retina with a differentiated GCL (ganglion cell layer), an INL (inner nuclear layer), and an ONL (outer nuclear layer) (indicated by the red arrows). (**B4**) Average number of GCL cells per section/per embryo in each group (*n* > 3, 3–5 sections from each embryo were used for counting the GCL cells). **C** Expression of retinal marker genes *gnat2*, *grk1b*, *grk7a*, and *opnlmw1* in embryos from control, Cu^2+^-stressed, and CuNPs-stressed groups. **D** WISH data of *gnat2* in embryos from control, Cu^2+^-stressed, and CuNPs-stressed groups, respectively (**D1**-**D3**), and the percentage of embryos exhibiting reduced expression in different groups (**D4**). **E** Expression of retinal marker genes *opn1sw1*, *opn1sw2*, *opn1lw1*, *rhodopsin*, *brn3b*, and *vsx1* in embryos from control, Cu^2+^ − stressed, and CuNPs-stressed groups. **F** WISH data of *opn1sw2* in embryos from control, Cu^2+^-stressed, and CuNPs-stressed groups, respectively (**F1-F6**), and the percentage of embryos exhibiting reduced expression in different groups (**F7**). **B1-B3**, sagittal slides in eyes domain; **D1-D3**, dorsal view, anterior to the left; **F1**-**F3**, dorsal view, anterior to the up; **F4**-**F6** lateral view, anterior to the left. Scale bar: **A**, **B1-B3**, and **F1-F6**, 100 μm; **D1-D3**, 200 μm **, *P* < 0.01; *, *P* < 0.05

The retinal diameter of the copper stressed embryos was measured at different developmental stages in this study first. As illustrated in Fig. 1A, both CuNPs and Cu^2+^ stressed embryos exhibited a reduced eye diameter from 72 hpf onward (Fig. 1A). Zebrafish retina contains three layers of cells, including ONL (outer nuclear layer), INL (inner nuclear layer), and GCL (ganglion cell layer) [37]. H&E staining of retinal sections demonstrated reduced cell number in the GCL of copper-treated zebrafish at both 72 hpf and 96 hpf (Fig. 1B and Fig. S1).

Based on the RNA-seq results for CuNPs or Cu^2+^ stressed embryos we reported recently [18], this study showed that both CuNPs and Cu^2+^ down-regulated the expression of the genes associated with the development of eyes (Fig. S2 and Table S2). Next, the expression of four optic nerve related genes (*gnat2, grk1b, grk7a, opn1mw1*) was measured in copper stressed and the control embryos by qPCR in this study. All the expressions of these genes were found to be reduced in copper stressed embryos (Fig. 1C). Down-regulated expression of *gnat2* in 72 hpf copper stressed embryos was observed by WISH (Fig. 1D).

WISH and qPCR were performed further to test the initiation of eye development and the specification and differentiation of retinal cone and rod cells in copper stressed embryos. The progenitor markers of eyes such as *pax2a*, *otx2*, *rx1*, and *rx2* exhibited down-regulated expression in copper stressed embryos (Fig. S3). Additionally, the expressions of cone cell marker (*opn1sw2*) (Fig.1E and F**)** and cone cell markers (*opn1sw1* and *opn1lw1*) and one rod cell marker (*rhodopsin*) (Fig. 1e and S4) were all reduced in copper-stressed embryos. The retinal *vsx1* (INL layer marker) and *brn3b* (GCL layer marker) also showed decreased expression in copper-stressed zebrafish (Fig. 1E).

**Fig. 2:**
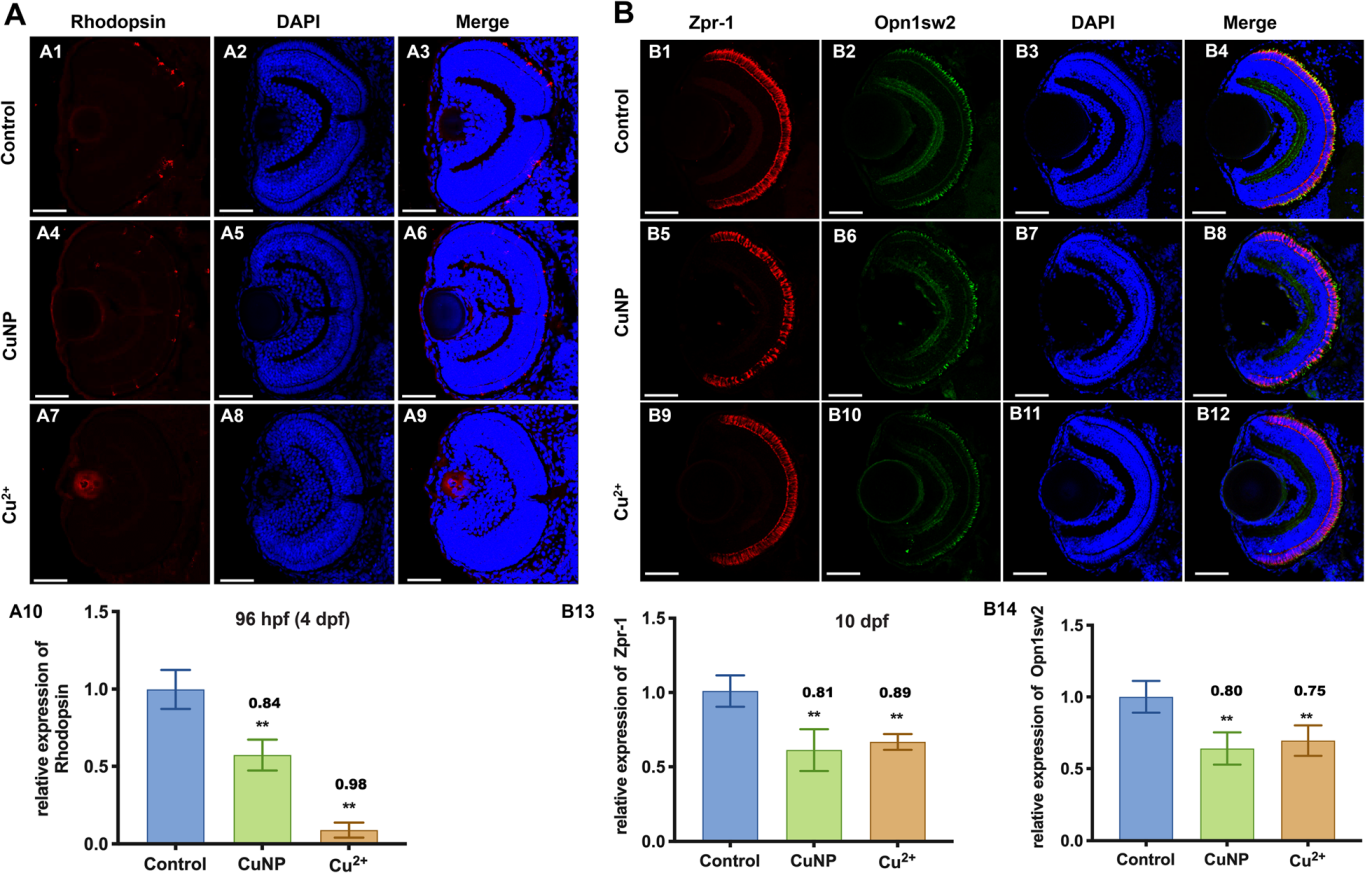
Protein levels of retinal photoreceptor in copper stressed embryos. **A** Immunostaining of Rodopsin (labeling retinal rod cells) (**A1-A9**). **B** Double immunostaining of ZPR-1 (labeling retinal cone cells) and Opn1sw2 (blue opsin, labeling retinal rod cells) in embryos at 10 dpf. **A10**, **B13**, **B14**, Quantification of protein level in each sample. Scale bars: **A1**-**A9** and **B1**-**B12,** 50 μm; **, *P* < 0.01; *, *P* < 0.05

Moreover, a decrease was observed in photoreceptor cells (ONL layer) in copper-treated embryos at both 96 hpf and 10 dpf by immunofluorescence analysis with Rhodopsin (marker of rod cell outer segment), Zpr-1 & Zpr-3 (marker of the double cones’ cell bodies), and Opn1sw2 & Opn1lw1 (blue and red opsin respectively, marker of blue or red cone outer segment) (Fig. 2A, B**,** S5, and S6).

### Cell proliferation and apoptosis in copper stressed embryos

**Fig. 3:**
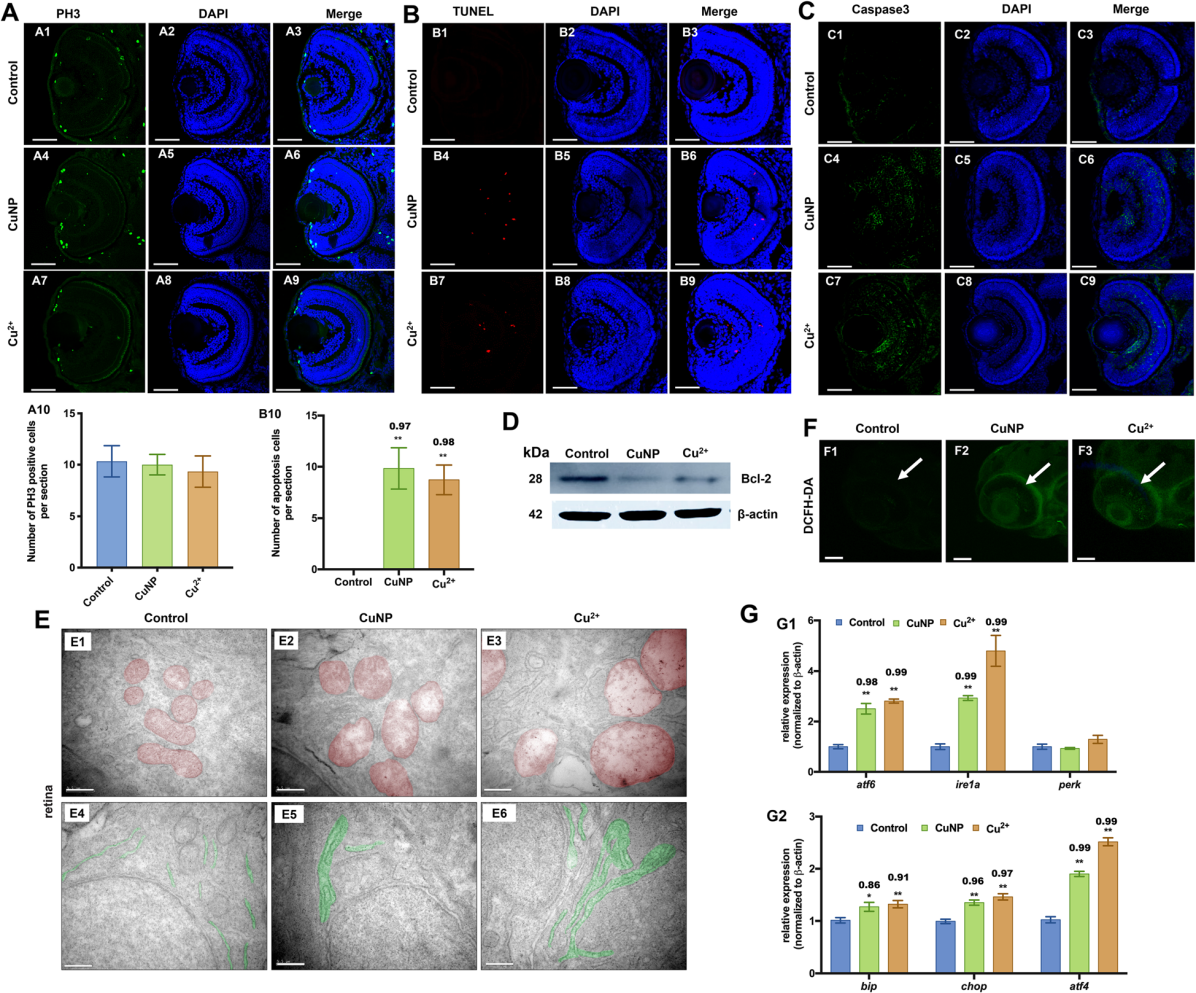
Cell proliferation, apoptosis, and oxidative & ER stresses in copper-stressed embryos. **A** Cell proliferation assay by PH3 staining (green dots). **B** Cell apoptosis assay by TUNEL (red dots) detection. **(A, B)** sections of embryonic eyes. **A10**, number of PH3 positive cells per section, **B10**, average number of apoptotic cells of retinal sections in each group (*n* > 3, 3–5 sections from each embryo were used for counting the red positive apoptotic cells). **C** Immunostaining of Caspase3 (green) in retina sections. **D** Western blot detection with antibody Bcl-2 in copper-stressed embryos. **E** TEM analysis of retinal cells in copper-stressed embryos at 96 hpf. **E1**-**E6**: sagittal slides of retina, red color indicating mitochondria and green color indicating ER. **F** DCHF-DA assay of embryonic retina. **G** qRT-PCR detection of ER-stressed genes in embryos at 96 hpf. Scale bars: **A1**-**A9, B1**-**B9 and C1-C9**, 50 μm; **E1**-**E6**, 0.5 μm; **F1-F3,** 100 μm; **, *P* < 0.01; *, *P* < 0.05

In order to unveil the mechanism of the decrease in photoreceptor cells occurred in copper stressed embryos, we tested cell proliferation or apoptosis in embryonic retina next. Phosphorylation at Ser10 of histone H3 is tightly correlated with chromosome condensation during both mitosis and meiosis [38]. Results in this study showed that the number of phospho-H3-positive nuclei exhibited no significant change following copper treatment in retinal cells at 72 hpf (Fig. 3A). However, TUNEL assay and increased level of Caspase3 indicated the activation of apoptosis in copper-treated embryonic retina (Fig. 3B and C). Moreover, Western-blot showed the down-regulated expression of Bcl-2 protein, a well-known negative regulator of apoptosis [39] in copper-treated embryos (Fig. 3D).

### Copper induces ROS and ER stresses in embryonic cells

ROS and ER stresses are principle triggers in initiating apoptosis [40, 41], and copper has been unveiled to induce ROS and ER stresses in in vitro cells [4]. We therefore measured ROS and ER responses in copper stressed embryos. TEM analysis showed that the ER and mitochondria structures were disrupted in copper-treated embryonic retinal cells (Fig. 3E). Compared with the control, the mitochondrial inner membrane was found to decrease and produce large vacuoles (Fig. 3E1-E3**,** pseudocolored in red), and ER formed loose structures (Fig. 3E4-E6**,** pseudocolored in green) in copper stressed retinal cells. Meanwhile, DCFH-DA assays indicated increased ROS occurred in copper stressed embryonic retina (Fig. 3F). Furthermore, the expressions of ER stress associated genes were tested in copper-treated embryos by qPCR. The expression remained unchanged in *perk*, but was up-regulated in *ire1α* and *atf6* at 96 hpf (Fig. 3G1). Additionally, the expression of *atf4*, a downstream gene of *perk*, and two ER stress markers (*bip* and *chop*) was increased after copper treatment (Fig. 3G2).

**Fig. 4:**
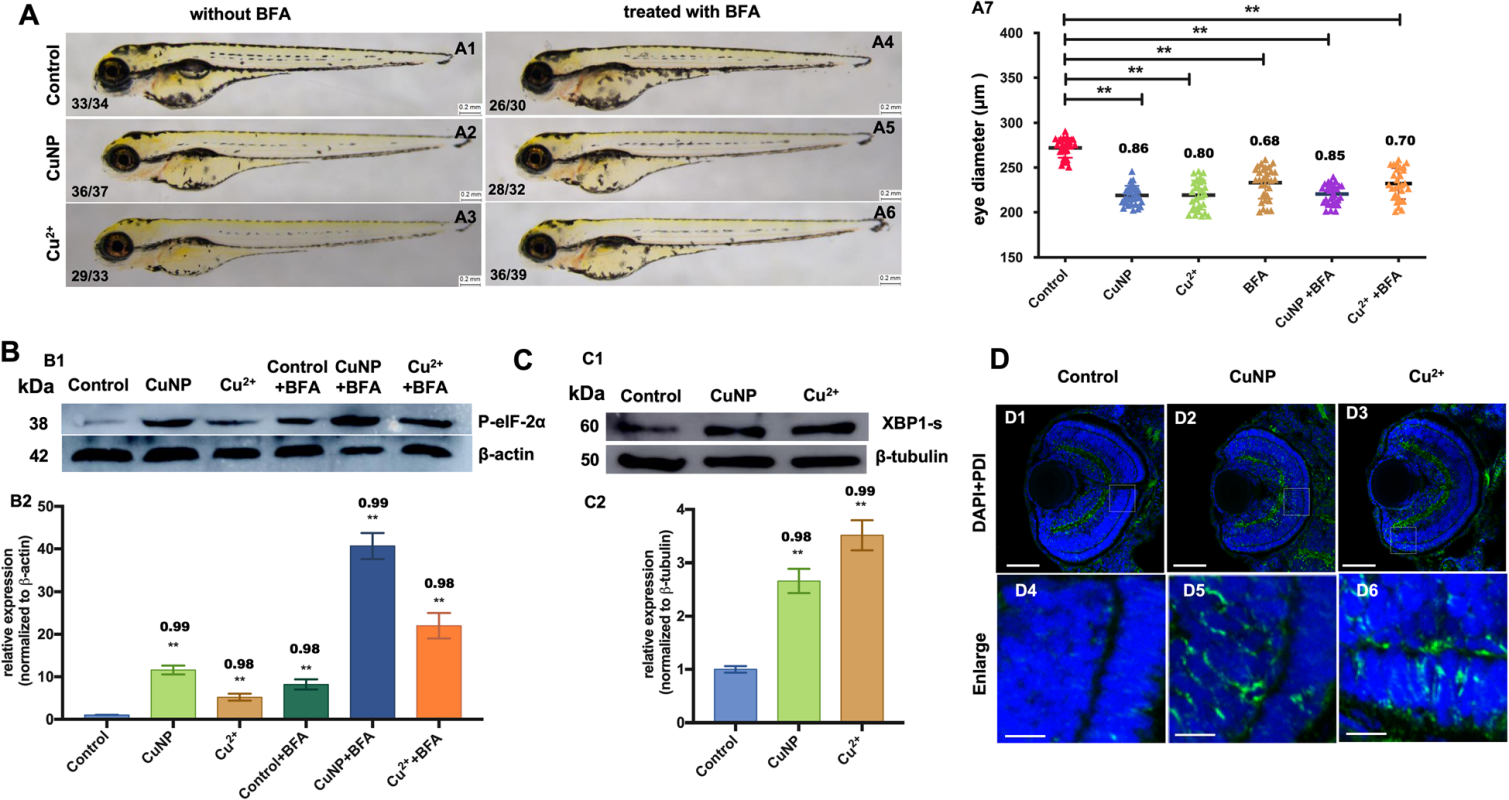
ER stresses in copper-stressed and BFA-treated embryos. **A** Phenotypes of representative copper-stressed embryos with or without BFA. BFA treatment destroys the COPII function and induces ER stresses, **A7,** measurement of eye diameters of the embryos. **A1**-**A6**, lateral view, anterior to the left. **B** Protein levels of P­eIF-2α in copper-stressed embryos with or without BFA (**B1**) and the quantification of protein levels of P­eIF-2α in each sample (**B2**). **C** Western blot detection of XBP1-s in copper-stressed embryos (**C1**) and the quantification of protein level in each sample (**C2**). **D** Immunostaining of PDI (ER marker) in copper-treated embryos. **D1**-**D3,** merged images of immunostaining of PDI (green) and DAPI staining (blue); **D4**, **D5** and **D6** were magnified domains of white boxes marked in **D1, D2** and **D3,** respectively. **D1- D6**, sagittal slides in eyes domain. Scale bar: **A1-A6**, 0.2 mm; **D1**-**D9,** 50 μm; **D10**-**D12,** 10 μm. **, *P* < 0.01; *, *P* < 0.05

BFA (Brefeldin A) is a commonly used protein transport inhibitor specifically blocking protein transport from ER to Golgi apparatus [42, 43]. A similar microphthalmia phenotype was observed in copper-treated embryos, just like the case of BFA-treated embryos (Fig. 4a). It was reported that Perk-phosphorylated eIF2α resulted in the attenuation of protein synthesis [44], and Ire1α cleaved XBP1 mRNA to form spliced XBP1, leading to ER-associated degradation proteins [45]. Based on these findings, this study evaluated the protein expression levels of p-eIF2α and XBP1s (spliced XBP1) in copper-treated embryos. Consistently, our results unveiled that the expression of p-eIF2α was increased in BFA treated embryos (Fig. 4b), but it was higher in copper and BFA co-treated embryos than in copper-treated embryos (Fig. 4B). Additionally, our results indicated that copper increased levels of XBP1s in copper stressed embryos (Fig. 4C). Immunostaining with a PDI (misfold protein marker) antibody was used to evaluate the misfold protein in ER and an increased PDI level was observed in copper-treated embryonic retinal cells (Fig. 4D).

### Scavenging ROS and relieving ER stress can recover retinal defects in copper stressed embryos

**Fig. 5:**
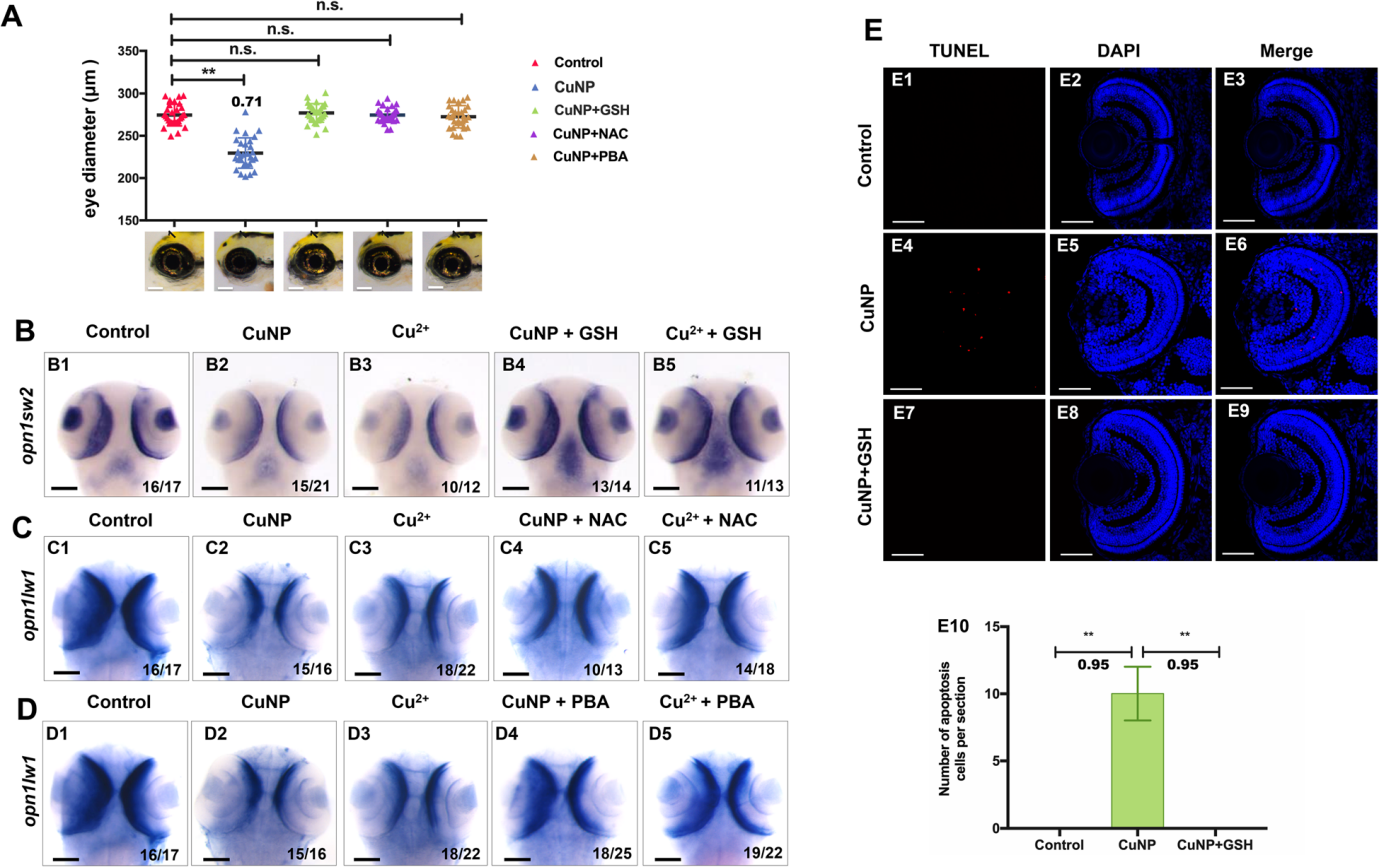
Rescue of retinal defects with the addition of ROS scavenger and ER stress scavenger. ROS scavengers GSH & NAC and ER scavenger PBA recovered the copper induced small eye defects (**A**) and the expression of retinal markers *opn1sw2* (**B1**-**B5**) & *opn1lw1* (**C1-C5** and **D1-D5**) in copper-stressed embryos. **E** ROS scavengers GSH significantly neutralized copper induced cell apoptosis in embryos. Average number of apoptotic cells of retinal sections in each group **(E10)** (*n* > 3, 3–5 sections from each embryo were used for counting the red positive apoptotic cells). **B1**-**B5**, **C1**-**C5**, and **D1**-**D5**, dorsal view, anterior to the up; **E1- E9**, sagittal slides in eyes domain. Scale bar, **A, B1**-**B5**, **C1**-**C5**, and **D1**-**D5**, 100 μm; **E1-E9**, 50 μm. **, *P* < 0.01; *, *P* < 0.05

Next, we found that two kinds of ROS scavenger (NAC and reduced GSH) and an ER stress inhibitor (PBA), could rescue copper-caused microphthalmos in this study (Fig. 5A). WISH data showed that the expressions of *opn1sw2* (blue opsin) (Fig. 5B1-B5) was restored to nearly normal levels by GSH supplementation in copper-treated embryos. Similarly, the expression of *opn1lw1* (red opsin) was recovered to normal level in copper stressed embryos via NAC or PBA supplementation (Fig. 5C1-C5, and Fig. 5D1-D5). TUNEL analysis indicated that retinal cell apoptosis was significantly recovered in copper stressed embryos via GSH supplementation (Fig. 5E1-E10).

### Retina development in copper stressed *cox17* and *atp7a* mutants

**Fig. 6:**
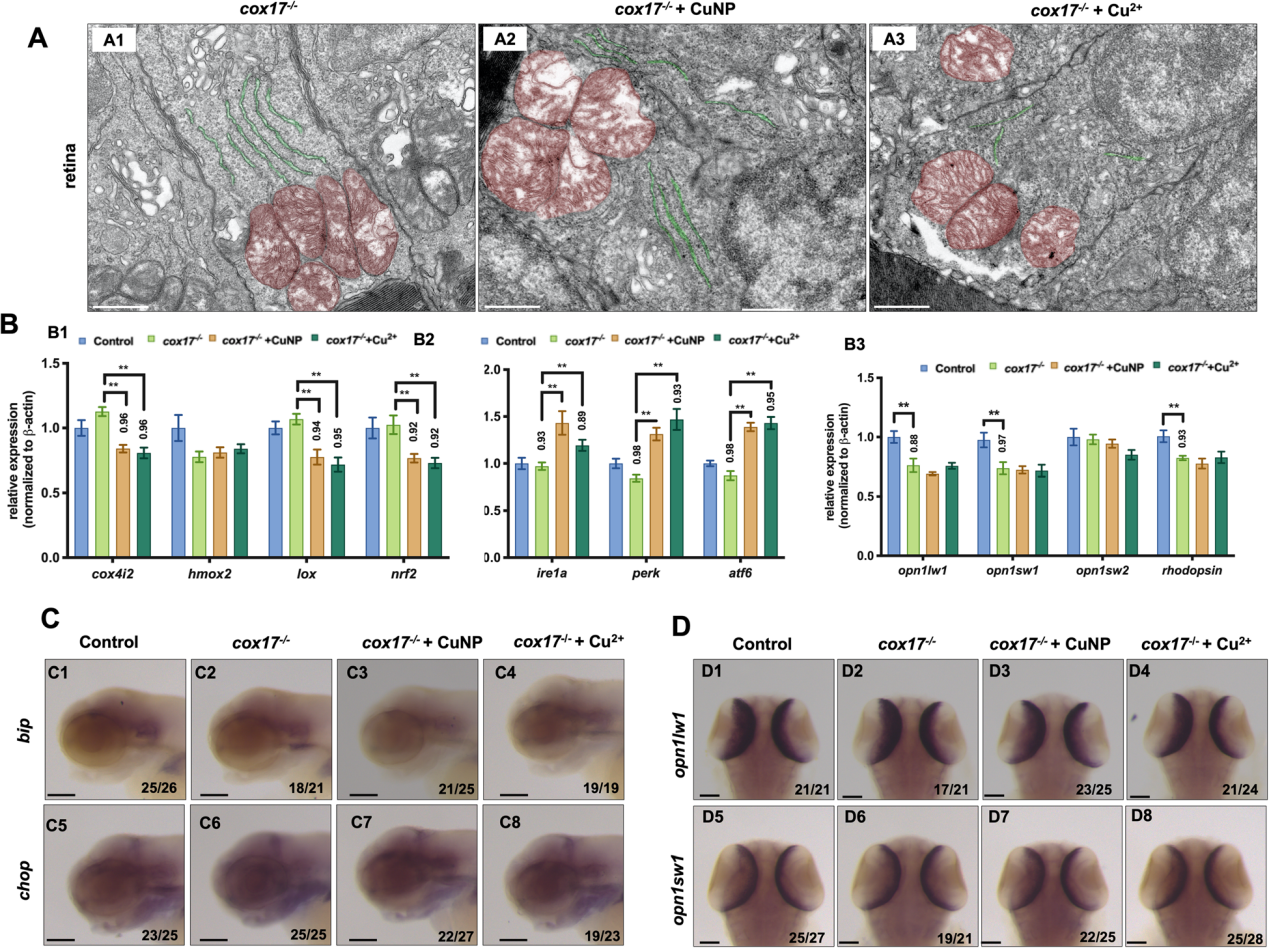
Retina development in *cox17*^*−/−*^ mutant treated with copper. **A** TEM analysis of retinal cells in copper stressed *cox17*^*−/−*^ mutant embryos at 96 hpf. **A1**-**A3**, sagittal slides of retina, red color indicating mitochondria and green color indicating ER. **B** Quantitative PCR of ROS related genes (**B1**; *cox4i2*, *hmox2*, *lox*, and *nrf2*), UPR related genes (**B2**; *ire1a*, *perk*, and *atf6*), and retinal genes (**B3**; *opn1wl1*, *opn1sw1, opn1sw2 and rhodopsin*) in *cox17*^*−/−*^ mutants. **C** WISH assays of ER stress marker *bip* and *chop* in *cox17*^*−/−*^ mutants. **D** WISH assays of retinal marker genes (*opn1wl1* and *opn1sw1*) in *cox17*^*−/−*^ mutants. **C1**-**C8,** lateral view, anterior to the left, **D1**-**D8**, dorsal view, anterior to the up, and Scale bar: **A1**-**A3,** 1 μm; **C1-C8** and **D1-D8**, 100 μm. **, *P* < 0.01; *, *P* < 0.05

Then, we asked the integral roles of copper transporting protein, Cox17 and Atp7a, in copper induced embryonic retinal defects in this study. A *cox17* mutant (4 bp deletion, being constructed in our lab) [35] was used to test the default of copper trafficking to mitochondrion in retinal developmental defects in copper stressed embryos. In *cox17*^*−/−*^ mutants, normal-like mitochondria and ER structures were observed in retinal cells (Fig. 6A1). After copper treatment, slightly swollen mitochondria (Fig. 6A2 and A3, pseudocolored in red) and normal-like ER (Fig. 6A2 and A3**,** pseudocolored in green) were observed in retinal cells. Expressions of ROS and ER related genes were tested further in *cox17*^*−/−*^ mutants under copper or no copper treatment. Among the four tested redox-related and antioxidant genes, the expression of *cox4i2, lox*, *hmox2*, and *nrf2* exhibited almost no significant change in *cox17*^*−/−*^ mutants when compared with WT (wild type) embryos (Fig. 6B1). After either CuNPs or Cu^2+^ treatment, the expressions of *cox4i2, lox*, and *nrf2* were significantly down-regulated in the mutants (Fig. 6B1). Quantitative PCR revealed no change in the expression of all the tested UPR sensors (*ire1a, atf6*, and *perk*) in *cox17*^*−/−*^ embryos under no copper treatment with increased expression in the corresponding genes after either CuNPs or Cu^2+^ treatment (Fig. 6B2). Moreover, CuNPs induced increased expression of *bip* and *chop* in brain in WT embryos (Fig. S7A2 and A6), but in *cox17*^*−/−*^ mutants, unchanged expression of *bip* and *chop* in the brain (Fig. 6C3, C4, C7, C8 and S7A) was observed after either CuNPs or Cu^2+^ treatment.

Significantly reduced expression of retinal cell markers (*opn1lw1, opn1sw1,* and *rhodopsin*) was revealed by qPCR detection in the whole *cox17*^*−/−*^ mutant embryos (Fig. 6B3). However, the expression remained unchanged in the aforementioned genes in the mutants after either CuNPs or Cu^2+^ treatment (Fig. 6B3). Additionally, WISH unveiled a little reduced expression of retinal marker *opn1lw1* (Fig. 6D2) and *opn1sw1* (Fig. 6D3) in *cox17*^*−/−*^ mutants, while their expression remained unchanged (Fig. 6D3, D4, D7, D8 and S7B) after either CuNPs or Cu^2+^ treatment.

**Fig. 7:**
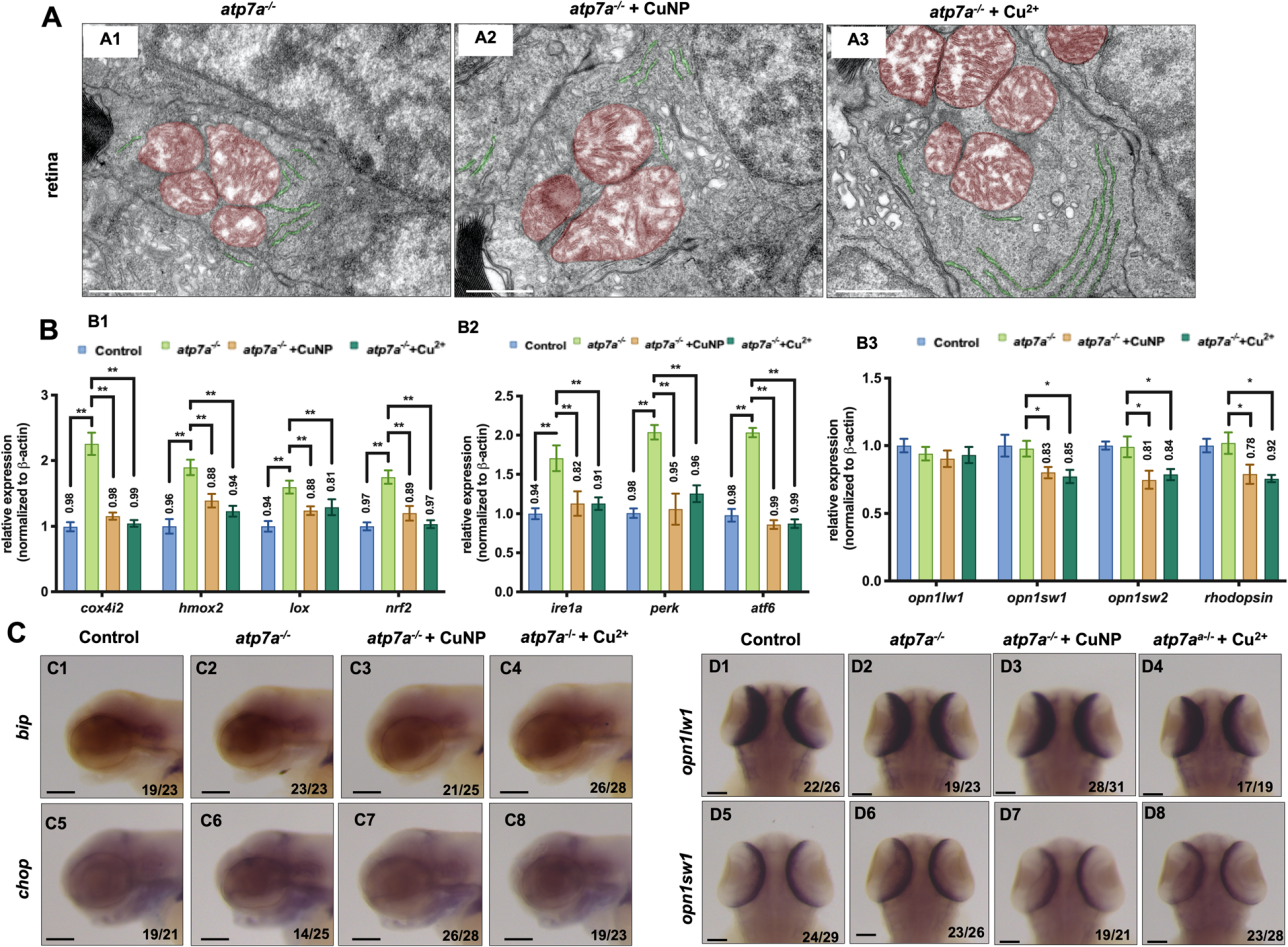
Retina development in *atp7a*^*−/−*^ mutant treated with copper. **A** TEM analysis of retinal cells in copper-stressed *atp7a*^*−/−*^ mutant embryos at 96 hpf. **A1**-**A3**, sagittal slides of retina, red color indicating mitochondria and green color indicating ER. **B** Quantitative PCR of ROS related genes (**B1**; *cox4i2*, *hmox2*, *lox*, and *nrf2*), UPR related genes (**B2**; *ire1a*, *perk*, and *atf6*), and retinal genes (**B3**; *opn1wl1*, *opn1sw1, opn1sw2 and rhodopsin*) in *atp7a*^*−/−*^ mutants. **C** WISH assays of ER stress marker *bip* and *chop* in *atp7a*^*−/−*^ mutants. **D** WISH assays of retinal marker (*opn1wl1* and *opn1sw1*) in *atp7a*^*−/−*^ mutants. **C1**-**C8**, lateral view, anterior to the left, **D1**-**D8**, dorsal view, anterior to the up. Scale bar: **A1**-**A3,** 1 μm; **C1-C8** and **D1-D4**, 100 μm; **, *P* < 0.01; *, *P* < 0.05

In *atp7a*^*−/−*^ mutants (being constructed in our lab, its mutation information and developmental phenotypes, transcriptional profiles, were detailed in another manuscript), TEM analysis revealed slightly swollen mitochondria and normal-like ER (Fig. 7A1, mitochondria was pseudocolored in red and ER was pseudocolored in green**)** in *atp7a*^*−/−*^ retinal cells. After copper treatment, the structures of mitochondria and ER remained unchanged in *atp7a*^*−/−*^ retinal cells (Fig. 7A2 and A3). The expressions of redox-related and antioxidant genes (Fig. 7B1) and UPR sensors (Fig. 7B2) were increased in the *atp7a*^*−/−*^ mutants when compared with the WT control (Fig. 7B1 and B2). However, their expression in the mutants was significantly down-regulated after treatment with either CuNPs or Cu^2+^ (Fig. 7B1 and B2). WISH detections also revealed notably reduced expression of ER genes *bip* and *chop* in either CuNPs or Cu^2+^ treated mutants (Fig. 7C3, C4, C7, C8 and Fig. S8A4, A8) compared with the untreated mutants (Fig. 7C2, C6 and Fig. S8A3, A7), although CuNPs elevated both *bip* and *chop* expression in brain in WT embryos (Fig. S8A2 and A6).

Normal expression of retinal markers (*opn1sw1, opn1sw2, opn1lw1,* and *rhodopsin*) was unveiled in untreated *atp7a*^*−/−*^ mutants by both qPCR and WISH detections (Fig. 7B3, d and Fig. S8B). After either CuNP or Cu^2+^ treatment, the expression showed a slight reduction in genes *opn1sw1, opn1sw2,* and *rhodopsin* (Fig. 7B3, D and Fig. S8B) in *atp7a*^*−/−*^ mutants.

## Discussion

### Copper induce retinal developmental defects in zebrafish embryos via up­regulating ROS and ER stress in embryonic cells

The toxicity of copper to vertebrate development and growth has been widely investigated recently [46–48]. However, little is discussed the copper induced retinal developmental defects and the potential mechanisms in an in vivo vertebrate model. In this study, embryos stressed with 3.9 μM copper were found to have small eyes, and the molecular characteristics of the microphthalmia defects were examined by qPCR, WISH, H&E staining, and immunofluorescence. The defects of small eyes were observed in *sec13/sec31* and *sec23* mutants [49, 50]. In this study, similar molecular expression patterns of retinal marker genes were observed in copper-stressed embryos, just as in zebrafish *sec13* mutants with retinal defects [21], suggesting that extracellular CuNPs and Cu^2+^ might affect the development of both retinal system by damaging the expression and regulation of intracellular genes and signals, similar to the behavior of *sec* family genes in zebrafish embryos.

Studies have shown that the copper content in WD brain reaches 125 μg/g [51], and the copper concentration in WD liver was over 40 μg/g [52]. However, in our recent reports [19, 34], nearly 10 μg/g copper at 24 hpf (hours post fertilization) and 2–8 μg/g copper at 48 hpf were detected in both 3.9 μM Cu^2+^ and CuNPs treated embryos. Compared with the copper concentration in WD liver and brain, the relatively far lower concentration in cells of copper treated embryos might not be excess to induce non-physiological responses, and the copper stressed zebrafish model in this study should be suitable genetic models for human disease with overload copper.

This study revealed that swollen & vacuolar mitochondria and cracked ER were observed in copper stressed retinal cells, suggesting the damaged mitochondria and ER occurred in the cells. The observations in this study are consistent with that cells exhibited damaged mitochondria and ER structure and increased ER and oxidative stresses under some simulations [4, 53]. Additionally, increased ROS has been reported in copper stressed embryos [19], zebrafish ZFL cells [26], and isolated liver mitochondria [25]. ROS and its derivatives from the redox status change can damage brain cells, leading to neurodegenerative diseases [54, 55].

In copper stressed embryos, up-regulated expression was observed in ER sensor genes such as *atf6* and *ire1a* as well as PDI, a chaperone in ER, which can mark misfolded protein [56–58], and whose increase in cells usually indicates the accumulation of misfolded proteins, resulting in ER stress in cells [27]. Moreover, copper-stressed embryos exhibited an increased expression in p­eIF-2α and a small eye phenotype, just like the case in BFA stressed embryos. BFA was reported to be a commonly used protein transport inhibitor that specifically blocks protein transport from ER to Golgi apparatus [59, 60], and the failure of protein transport would lead to ER stress in cells [43].

In this study, both ROS scavenger GSH&NAC and ER stress antagonist (PBA) effectively rescued microphthalmia defects caused by excess copper, and PBA recovered the mis-regulated expression of retinal genes to almost normal level in copper stressed embryos. It has been unveiled that both ER and oxidative stresses have a synergistic effect on tissue damage [61]. Our aforementioned results suggest that copper might induce the retinal developmental defects via increasing both ER and ROS stresses in copper stressed retinal cells, similarly as our current reports that copper significantly upregulated ROS to inhibit erythropoiesis and hatching during zebrafish embryogenesis [19, 62].

### ROS and ER stresses mediate copper induced retinal defects by triggering cell apoptosis

In the study, apoptosis was detected in copper stressed embryonic retina by TUNEL assay and increased expression of Caspase3. ROS and ER stresses were both reported to induce apoptosis [40, 41]. ROS triggered apoptosis mainly through the IRE1α-ASK1-JNK pathway [63]. ER stress-induced apoptosis pathways mainly include CHOP pathway, IRE1α-ASK1-JNK pathway, and Caspase-12 pathway. IRE1α-ASK1-JNK pathway inhibits the activity of the anti-apoptotic molecule BCL-2 and induces apoptosis [64]. Here, increased ROS was observed in retina in copper stressed embryos. Furthermore, both XBP1-s and p­eIF-2α, the indicators for ER stress and regulators in ER-induced apoptosis, exhibited increased expression in copper-stressed embryos in this study. Additionally, the expression was up-regulated in UPR genes (*atf6*, *ire1a*, and *perk* down-stream genes *bip* and *chop*) and CHOP pathway associated genes, but down-regulated in anti-apoptotic molecule BCL-2 in copper stressed embryos. This finding was consistent with previously reported results [4], and also suggested that copper might induce apoptosis through the aforementioned ER and ROS induced apoptosis pathways.

Reduced retinal core & rod cells were observed in copper stressed embryonic eyes. Normal proliferation and increased apoptosis were observed in cells of embryos from the copper-stressed group. Moreover, ROS scavengers GSH could significantly neutralize cell apoptosis in copper stressed embryos. The integrated data further confirm that copper causes cell apoptosis through both ER stress and oxidative stress, leading to retinal defects. The observations in this study is similar as the previous observations that ROS scavenge NAC&GSH could rescue the delayed hatching caused by oxidative stress in copper-stressed embryos [62].

### Copper induced retinal defects might depend on integral function of *cox17* and *atp7a*

Cox17 is known as a copper trafficking protein responsible for transporting copper to mitochondrion [65], and mitochondrion is the main organelle producing ROS in cells [66]. In this study, *cox17*^*−/−*^ mutants were used to test the assumption that the transport of copper to mitochondria can be blocked in the mutants under copper treatment. Consistently, unchanged expression of ROS indicators in copper-stressed *cox17*^*−/−*^ mutants suggests that the copper being transported to mitochondria might be not enough for the production of ROS in the stressed *cox17*^*−/−*^ mutants.

The inner segments of retinal photoreceptor cells in zebrafish were revealed to possess megamitochondria with diameters exceeding 2 μm [67, 68] and most mitochondria exhibit long and thin structures in normal mammalian inner segments of photoreceptors [69, 70]. Mega-mitochondria can usually be observed under pathological conditions [71]. In this study, copper induced no obvious retinal defects in the *cox17*^*−/−*^ mutants, suggesting the presence of mega-mitochondria in retinal cells which might be an important organelle in handling copper induced stresses and the resultant developmental defects. The presence of mega-mitochondria in retinal cells might also contribute to the normal-like mitochondria observed in *atp7a*^*−/−*^ mutants before and after copper treatment, although *atp7a*^*−/−*^ mutants were used in this study to test the assumption that the transport of copper to TGN and pumping to circulation are blocked. Additionally, the failure of pumping copper to circulation in *atp7a*^*−/−*^ mutants might result in deficient copper in retinal cells, thus the observation of normal-like development of retina in both copper and non-copper stressed *atp7a*^*−/−*^ mutants, which was consistent with the report showing that deficiency of copper in retina could not induce developmental defects in *atp7a*^*−/−*^ mutants [72]. The point raised in this study was consistent with the point that Atp7a is the major copper chaperone for loading copper into copper-dependent enzymes during Golgi processing [73] and pumping copper from intestinal cells into circulation [74].

ER stress and ER sensors were up-regulated in *cox17*^*−/−*^ mutants after copper treatment. These observations supported the assumption that copper-mitochondria trafficking and ROS production are blocked in *cox17*^*−/−*^ mutants, but not ER stress. In copper stressed *cox17*^*−/−*^ mutants, retinal cells showed slightly swollen mitochondria and no significant mis-regulation in ER sensors; meanwhile, retinal marker genes exhibited normal-like expression. These observations not only suggest that copper induces retinal developmental defects via triggering both ROS and ER stresses, but also suggest that copper induces retinal developmental dependent on the integral function of *cox17*^*−/−*^. However, it is still unknown why both ROS and ER sensors exhibit significantly down-regulated expression in both *atp7a*^−/−^ and cox17^−/−^ after copper stimulation. It is reported that moderately sustained UPR can maintain a normal intracellular environment [75] and silencing of *AtCOX17* in *Arabidopsis* induced reduced or delayed oxidoreductase responses to stress [76], the protective mechanisms might be activated in either *atp7a*^−/−^ or *cox17*^*−/−*^ mutants in this study. Additionally, it is still known why retinal genes exhibited slightly reduced expression but the structure of mitochondria and ER were normal likely in *cox17*^*−/−*^ mutants, we speculated that the damage of integral function of *cox17* might block another signaling which parallel to ROS and ER stresses signaling in regulating retinal development during vertebrate embryogenesis.

Although retinal degeneration and copper overload in brain has been unveiled in WD patients for long time, however, few studies link the copper overload with retinal degeneration in WD patients, and the studies of the potential mechanisms are scarce. This study for the first time demonstrated that copper overload in cells triggers ROS and ER stresses, then lead to retinal cell apoptosis during vertebrate embryogenesis. The defective embryos observed in this study might be used as suitable vertebrate disease models for linking copper overload with retinal degeneration, and the regulation signaling unveiled in this study might apply some hints for potential mechanism underlying retinal degeneration occurrence in WD patients, just like genes *CERKL*, *SEC23A*, and *SEC13* function conservatively in retinal development and degeneration [20–22].

## Conclusions

**Fig. 8:**
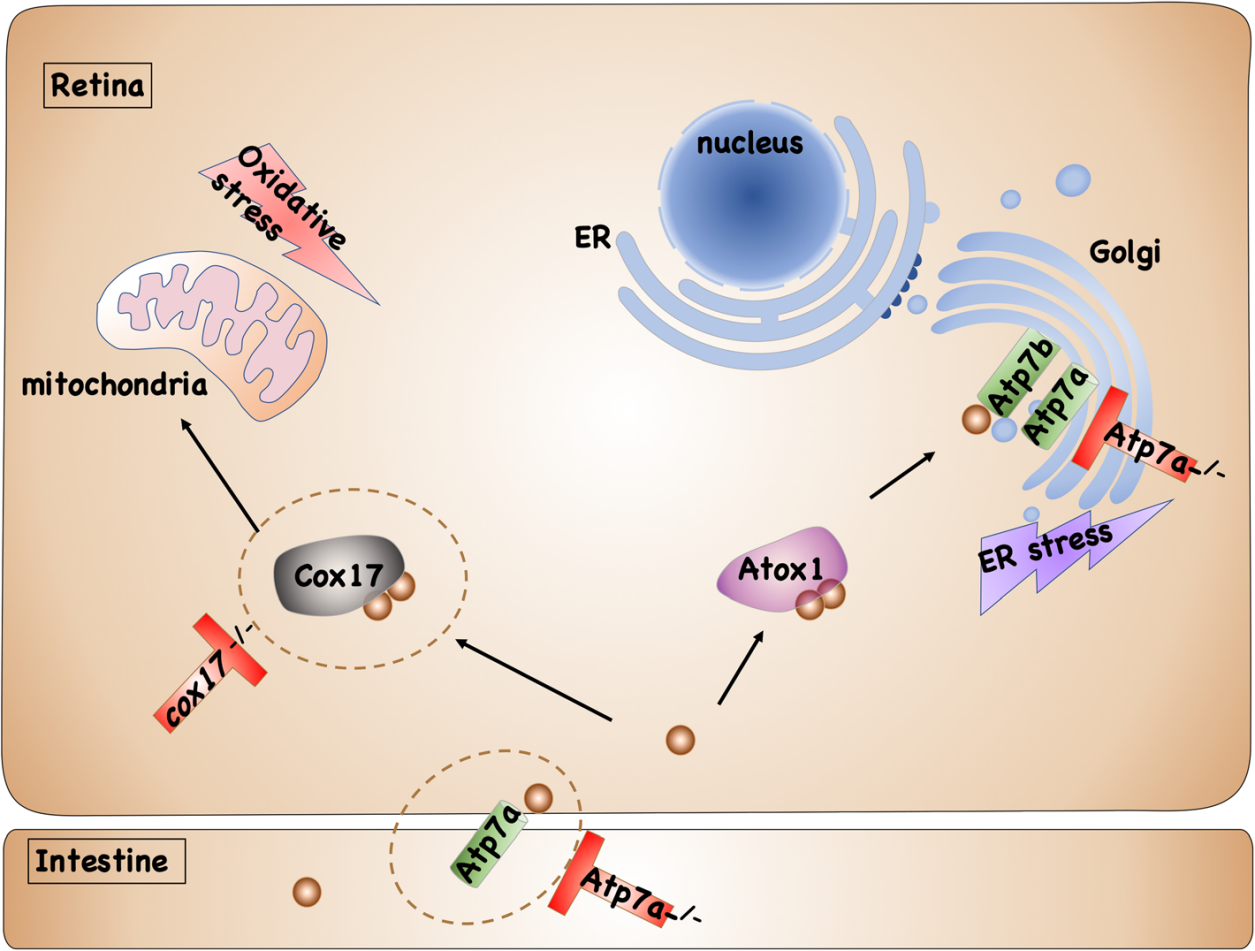
Model of copper induced retinal developmental defects in WT, *cox17*^*−/−*^, and *atp7a*^*−/−*^ embryos

This study made the first attempt to reveal the detailed molecular characteristics of copper induced retinal defects. Specifically, copper induces the apoptosis by triggering ROS and ER stresses, leading to developmental defects in embryonic retinal cells. The subtle correlation between different copper transporters and embryonic retinal defects was explored using *cox17*^*−/−*^ and *atp7a*^*−/−*^ mutants. For better understanding of this study, a simple schematic was drawn to show the transport of copper in the retinal cells and the functions of *cox17* & *atp7a* in copper induced retinal defects (Fig. 8). Moreover, the developmental regulating roles of copper overload in retinal defects unveiled in this study might shed some light on the studies linking copper overload in human cells with retinal degenerative diseases in future days.

## Supplementary information

**Additional file 1.** Supplementary materials include 6 supplementary figures and and 3 supplementary tables.

## Abbreviations

ADRP: Autosomal dominant retinitis pigmentosa
CuNPs: Copper_nanoparticles
dpf: Days post-fertilization
ED: Eales diseased
ER: Endoplasmic reticulum
GSH: Reduced Glutathione
hpf: Hours post-fertilization
MD: Menkes disease
NAC: *N*­acetylcysteine
PBA: 4-phenylbutyric acid
ROS: Reactive oxygen species
UPR: Unfolded protein responses
WD: Wilson disease
WT: Wild-type
