# Supplementary material for: Copper induce zebrafish retinal developmental defects via triggering stresses and apoptosis
Source: Cell Commun Signal. 2020 Mar 14;18:45. doi: 10.1186/s12964-020-00548-3 (PMC7071659; doi:10.1186/s12964-020-00548-3)
Supplement: Supplementary file 1 — Additional file 1. Supplementary materials include 6 supplementary figures and and 3 supplementary tables. [file 12964_2020_548_MOESM1_ESM.docx]

Table S1. qPCR and WISH primers used in this study

| **Gene** | **Full name** | **Primer (5’-3’)** | **For** |
| --- | --- | --- | --- |
| *opn1sw1(blue)* | *opsin 1, short-wave-sensitive 1* | F: TTTGGATGGAGCAGATAC  R: TGAGAAGAAAGCAGGGAT | qPCR and WISH |
| *opn1sw2(UV)* | *opsin 1, short-wave-sensitive 2* | F: CCTCGGGAACTTTACCTT  R: TTGGAAACCACCCAGATT | qPCR and WISH |
| *opn1lw1(red)* | *opsin 1, long-wave-sensitive 1* | F: TGAGGGTCCCAATTACCA  R: AGCAGATGCCCATTTAGC | qPCR and WISH |
| *rhodopsin(rod)* | *rhodopsin* | F: AACCTGGAGGGCTTCTTT  R: TATGACTAACAGTGGGATGA | qPCR and WISH |
| *rx1* | *retinal homeobox gene 1* | F: GTGCAGGTTTGGTTCCAG  R: CTCCAGAGGGTATTTGTCG | qPCR and WISH |
| *rx2* | *retinal homeobox gene 2* | F: TCGGGACGCATAAAGTGG  R: TGGTGGTCGGTTGAAGGA | qPCR and WISH |
| *otx2* | *orthodenticle homeobox 2a* | F: ACCGAAGGGCAAAGTGTC  R: GTCAAGTATGAGCCGCAGT | qPCR and WISH |
| *vsx1* | *visual system homeobox 1* | F: CGATGATGACTGCCTTTC  R: CATTCCTGCTGGCTGAC | qPCR |
| *brn3b* | *POU class 4 homeobox 1* | F: ATGAACCACAGGCACCAAG  R: GCGGCTGAATAGCAAAGTA | qPCR |
| *gnat2* | *guanine nucleotide binding protein*  *alpha transducing activity polypeptide 2* | F: ATGTTTGATGTGGGTGGC  R: ATGGAAGTTGTGGCGAAG | qPCR and WISH |
| *grk7a* | *G protein-coupled receptor kinase 7 a* | F: CAGGTGGACAGCAATGAA  R: GCTCAAACTTGGGCTCCT | qPCR |
| *grk1b* | *G protein-coupled receptor kinase 1 b* | F: CAGGTGGACAGCAATGAA  R: AAAGGTGGAGGAAGCATAC | qPCR |
| *opn1mw1* | *opsin 1, medium-wave-sensitive, 1* | F: GCATACCCGTAACCACAAT  R: GCAAACAAGAAGCCCAAA | qPCR and WISH |
| *ire1a* | *endoplasmic reticulum to nucleus*  *signaling 1* | F: ATGGCGTGGGGAGTGTGC  R: GTATTCTGTGCGGCCAAGGTAAA | qPCR |
| *atf6* | *activating transcription factor 6* | F: CTGGAGGCGCTGGTGAAAAGTG  R: CCGGACGGGAGATGGGAACA | qPCR |
| *perk* | *eukaryotic translation initiation factor 2-alpha kinase 3* | F: CCGCGGGGCAACAGAGT  R: GGTGGCAGCGATACAGAAGAAGAT | qPCR |
| *cox4i2* | *cytochrome c oxidase subunit 4I2* | F: AGGCAGATATGTCTCGACCAATG  R: TGTTCCAGGGCCCTTTCTCC | qPCR |
| *homx2a* | *heme oxygenase 2a* | F: GTTAAGGACCACGCAGTGTTTG  R: GAAGGCGCTCTGGATGAATG | qPCR |
| *nrf2a* | *nuclear factor, erythroid 2-like 2a* | F: AGAATGGGTCCGAAACAGAGC  R: GAAGGCGCTCTGGATGAATG | qPCR |
| *lox* | *lysyl oxidase a* | F: TGGCAGCACAATGGCAAACT  R: GAGCTGTCGGTTCTCGTTGC | qPCR |
| *bip* | *heat shock protein 5* | F: ATCAGATCTGGCCAAAATGC  R: CCACGTATGACGGAGTGATG | qPCR and WISH |
| *chop* | *DNA-damage-inducible transcript 3* | F: ATATACTGGGCTCCGACACG  R: GATGAGGTGTTCTCCGTGGT | qPCR and WISH |
| *atf4* | *activating transcription factor 4a* | F: GAGCACACTGAGGTTCCAG  R: GCTGCGGTTTTATTCTGCTC | qPCR |

Table S2. Down-regulated genes in eyes for clustering

| **Gene** | **Full name** | **Control** | **CuNPs** | **Cu2+** |
| --- | --- | --- | --- | --- |
| *cyp2p8* | *cytochrome P450, family 2, subfamily P, polypeptide 8* | 1 | 0.15978457 | 0.335254128 |
| *cyp3a65* | *cytochrome P450, family 3, subfamily A, polypeptide 65* | 1 | 0.25535921 | 0.11345397 |
| *cyp7a1a* | *cytochrome P450, family 7, subfamily A, polypeptide 1* | 1 | 0.111028349 | 0.078323986 |
| *gnat2* | *guanine nucleotide binding protein alpha transducing activity polypeptide 2* | 1 | 0.353602407 | 0.270683528 |
| *gnb3b* | *guanine nucleotide binding protein (G protein), beta polypeptide 3b* | 1 | 0.461755164 | 0.22036645 |
| *gngt2b* | *guanine nucleotide binding protein (G protein), gamma transducing activity polypeptide 2b* | 1 | 0.486900876 | 0.422412666 |
| *grk1b* | *G protein-coupled receptor kinase 1 b* | 1 | 0.298044571 | 0.261784938 |
| *grk7a* | *G protein-coupled receptor kinase 7 a* | 1 | 0.29078001 | 0.372631489 |
| *guca1c* | *guanylate cyclase activator 1C* | 1 | 0.255500851 | 0.157785387 |
| *irbp* | *interphotoreceptor retinoid-binding protein like* | 1 | 0.318905305 | 0.075140733 |
| *mbpa* | *myelin basic protein a* | 1 | 0.423519411 | 0.268290523 |
| *nr1d1* | *nuclear receptor subfamily 1, group d, member 1* | 1 | 0.292822842 | 0.163078941 |
| *opn1mw1* | *opsin 1, medium-wave-sensitive, 1* | 1 | 0.30462368 | 0.303555033 |
| *opn1sw1* | *opsin 1, short-wave-sensitive, 1* | 1 | 0.342529465 | 0.224906303 |
| *opn1sw2* | *opsin 1, short-wave-sensitive, 2* | 1 | 0.356506429 | 0.32268088 |
| *pde6c* | *phosphodiesterase 6C, cGMP-specific, cone, alpha prime* | 1 | 0.372755101 | 0.277381024 |
| *pde6h* | *phosphodiesterase 6H, cGMP-specific, cone, gamma, paralog a* | 1 | 0.401285241 | 0.19513169 |
| *pdia2* | *protein disulfide isomerase family A, member 2* | 1 | 0.252367843 | 0.119719661 |
| *pls1* | *plastin 1 (I isoform)* | 1 | 0.206698525 | 0.198456401 |
| *prph2a* | *peripherin 2a (retinal degeneration, slow)* | 1 | 0.391016123 | 0.287819252 |
| *prph2b* | *peripherin 2b (retinal degeneration, slow)* | 1 | 0.405929192 | 0.282257731 |
| *pvalb8* | *parvalbumin 8* | 1 | 0.153307481 | 0.172082823 |
| *rhbg* | *Rh family, B glycoprotein (gene/pseudogene)* | 1 | 0.46888304 | 0.466782054 |
| *rho* | *rhodopsin* | 1 | 0.423607489 | 0.288735419 |
| *si:dkey-205h13.2* |  | 1 | 0.03607392 | 0.290266302 |
| *syt5a* | *synaptotagmin Va* | 1 | 0.359957878 | 0.235858255 |
| *zgc:73075* |  | 1 | 0.461115479 | 0.432720088 |


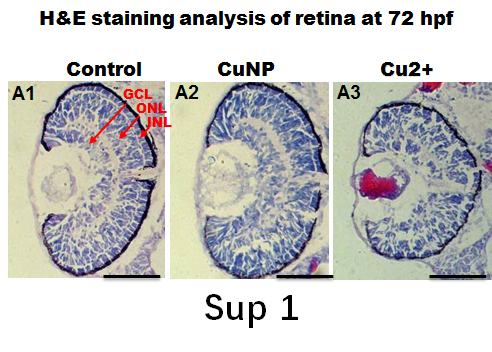


**Fig.S1** H&E staining analysis of retina of embryos from control (**A1**), CuNPs stressed (**A2**), or Cu^2+^ stressed group (**A3**) at 72 hpf. Scale bar, 100 μm.

**
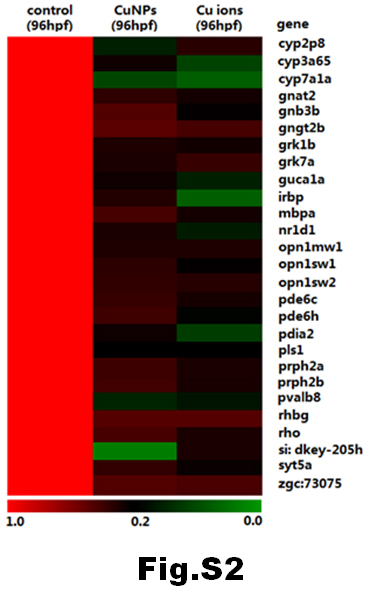
**

**Fig.S2** Down­regulated retinal genes in Cu^2+^ or CuNPs stressed embryos were screened by RNA-Seq assays and were clustered.


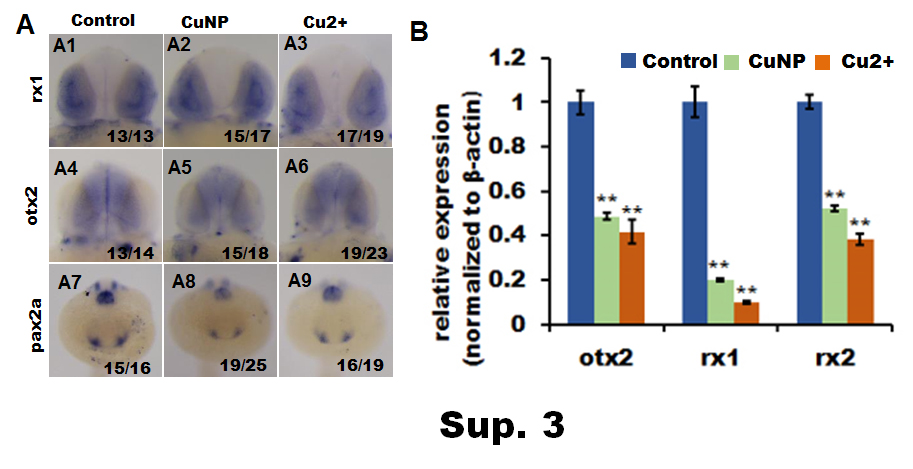


**Fig.S3** Down­regulated genes related to eyes progenitor cells in Cu^2+^ or CuNPs stressed embryos were detected by WISH (**A**) and qRT­ PCR (**B).** Scale bar, 100 μm.


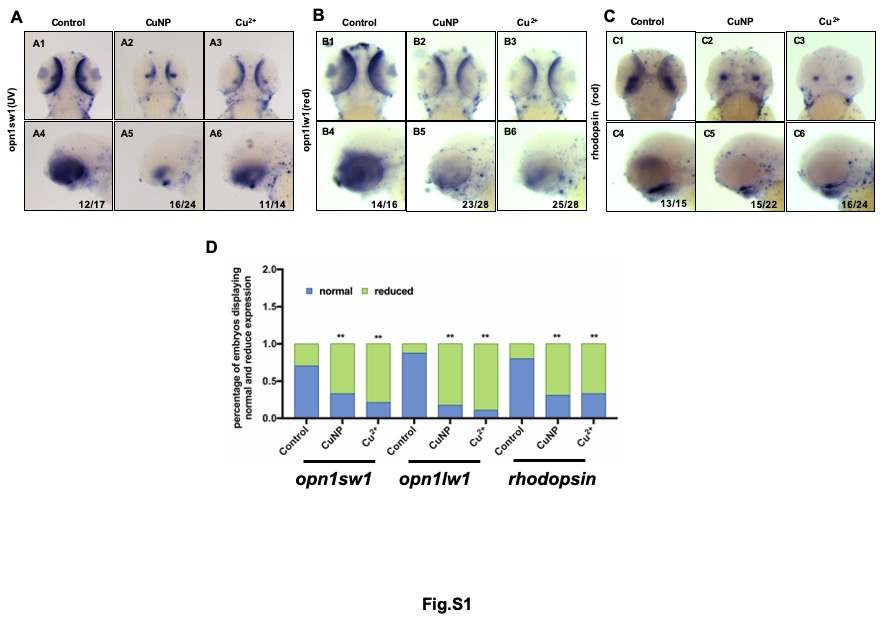


**Fig.S4** WISH data and the calculations and comparison of embryos exhibited reduced expression of *opn1sw2*, *opn1lw1*, and *rhodopsin* from different group. Scale bar, A-C,100 μm. ***P* < 0.01; *, *P* < 0.05.


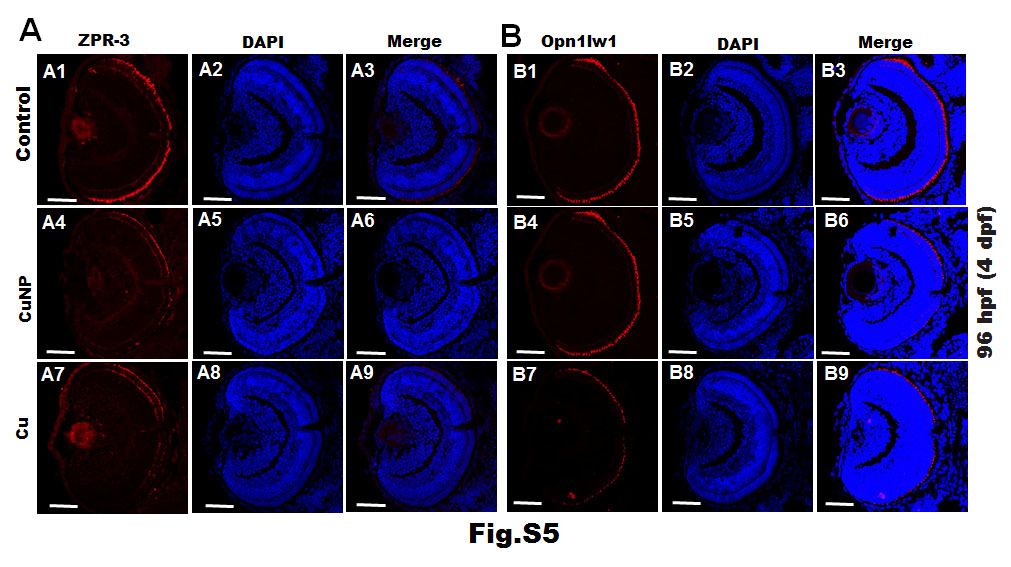


**B10**

**A10**

**
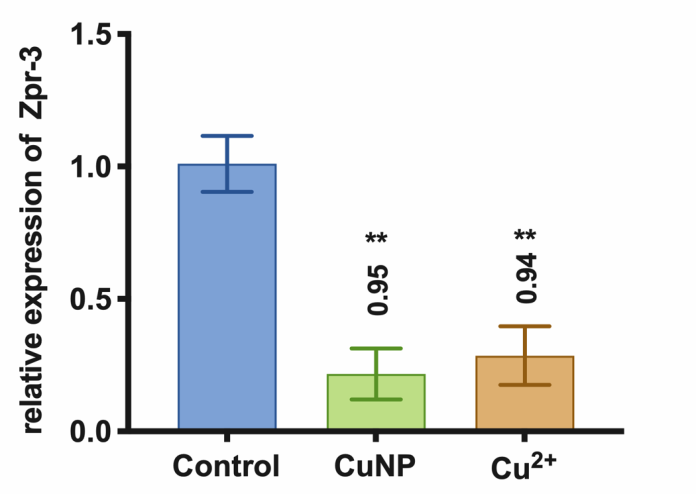

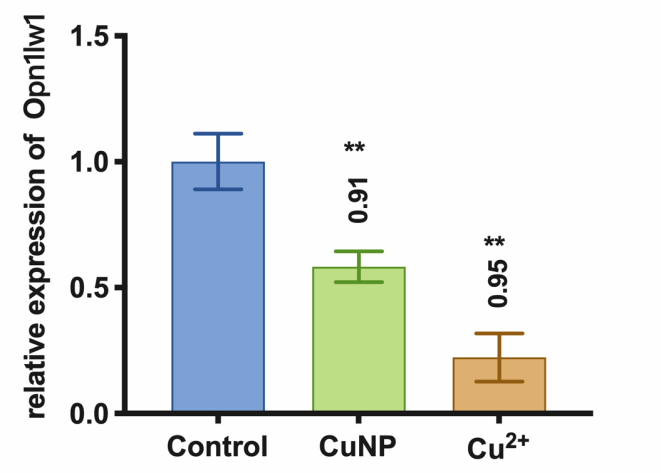
**

**Fig.S5** Immunostaining of ZPR-3 (labeling retinal cone cells) (**A**) and Opn1lw1(red opsin) (labeling retinal rod cells) (**B**) in embryos stressed with Cu^2+^ or CuNPs at 96 hpf (4dpf) respectively. Scale bar, 100μm.


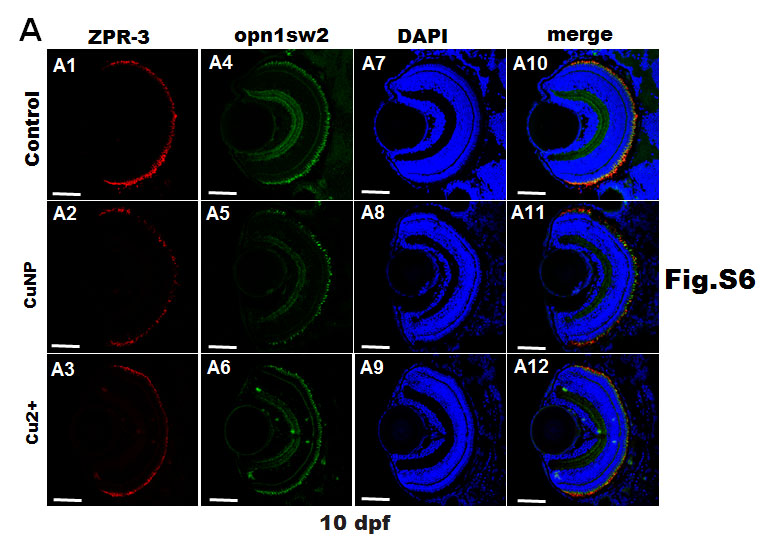

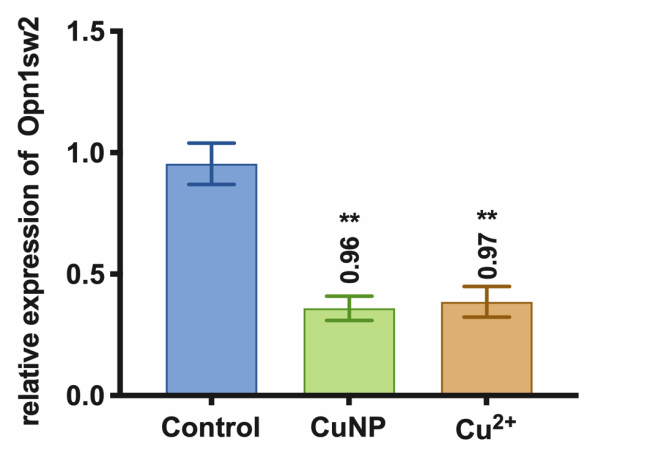
**
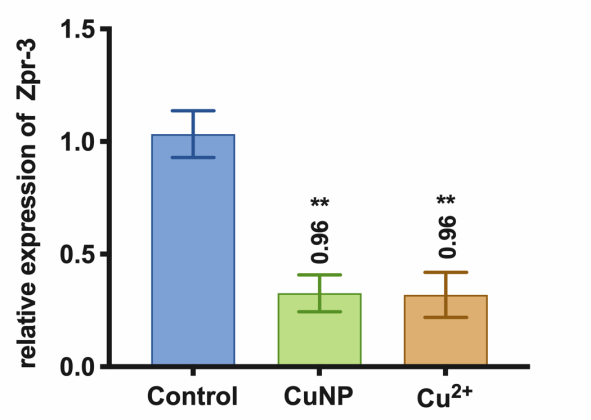
**

**A14**

**A13**

**Fig.S6** Immunostaining of ZPR-3 (red positive signals, labeling retinal cone cells) (**A**) and Opn1sw2 (blue, green positive signals, labeling retinal rod cells) in embryos stressed with Cu^2+^ or CuNPs at 10 dpf. Scale bar, 100μm.

**Fig.S7.** WISH data of *bip*, *chop*, *opn1lw1* and *opn1sw1* in WT and *cox17^-/-^* mutants

with or without CuNPs stimulation

**Fig.S8.** WISH data of *bip*, *chop* and *opn1sw2* in WT and *atp7a^-/-^* mutants with or without CuNPs stimulation
